# Innate immune function during antineoplastic treatment is associated with 12-months survival in non-small cell lung cancer

**DOI:** 10.3389/fimmu.2022.1024224

**Published:** 2022-12-12

**Authors:** Heidi Ryssel, Kristian Egebjerg, Susanne Dam Nielsen, Jens Lundgren, Mette Pøhl, Seppo W. Langer, Andreas Kjaer, Sisse Rye Ostrowski, Barbara Malene Fischer

**Affiliations:** ^1^ Department of Clinical Physiology and Nuclear Medicine, Copenhagen University Hospital, Rigshospitalet, Copenhagen, Denmark; ^2^ Department of Oncology, Rigshospitalet, Copenhagen University Hospital, Copenhagen, Denmark; ^3^ Cluster for Molecular Imaging, Department of Biomedical Sciences, University of Copenhagen, Copenhagen, Denmark; ^4^ Department of Infectious Diseases, Copenhagen University Hospital, Rigshospitalet, Copenhagen, Denmark; ^5^ Department of Clinical Medicine, University of Copenhagen, Copenhagen, Denmark; ^6^ Centre of Excellence for Health, Immunity and Infections (CHIP), Rigshospitalet, University of Copenhagen, Copenhagen, Denmark; ^7^ Department of Clinical Immunology, Copenhagen University Hospital, Rigshospitalet, Copenhagen, Denmark; ^8^ School of Biomedical Engineering and Imaging Sciences, Kings College London, London, United Kingdom

**Keywords:** immune, immunotherapy, response, NSCLC, chemotherapy

## Abstract

**Introduction:**

The immune system has proven to be a key player in the progression as well as containment of cancer with new treatment strategies based on immunotherapy targeting this interaction. Assessing immune function could reveal critical information about the immune response to therapeutic interventions, revealing predictive biomarkers for tailored care and precision medicine.

**Methods:**

We investigated immune function in 37 patients with inoperable non-small cell lung cancer (NSCLC) undergoing treatment with PD-L1 immune checkpoint inhibitor (ICI), chemotherapy (CT) or chemo-radiotherapy (CT/RT). Blood samples before (day 0) and during therapy (day 7, 21 and 80) were investigated by a standardized immunoassay, TruCulture®.

**Results:**

Outcomes revealed a developing innate immune response induced by both immunotherapy and chemotherapy. NSCLC-patients displayed evidence of chronic innate immune activation and exhaustion prior to treatment. This pattern was particularly pronounced during treatment in patients dying within 12-months follow-up. Compared to treatment with CT, ICI demonstrated a higher ex vivo-stimulated release of proinflammatory cytokines.

**Discussion:**

These preliminary findings may pave the way for tailored treatment and immune-monitoring.

## Introduction

Immunotherapy with checkpoint inhibitors of PD-1/PD-L1 monoclonal antibodies, has profoundly changed the treatment and efficacy in patients with non-small cell lung cancer (NSCLC) (1–4). Though, overall survival has improved with immune checkpoint inhibitor (ICI), many patients with NSCLC do not benefit from treatment or experience early progression due to primary or acquired resistance (2–4). Due to the complex interaction between the immune system and tumor biology, it is unlikely that a single biomarker, such as PDL-1 expression, will be sufficient to predict clinical outcomes in response to immune-targeted therapy (5). Further, treatment failure is often observed - even in patients with high PDL-1 expression (4). Effective biomarkers for ICI response could tailor care by selecting patients with a high likelihood of clinical response to treatment (2), and improving response monitoring and clinical outcome (2). Over the recent years, several different biomarkers have been evaluated in order to replace or complement PD-L1 predictivity, e.g. neoantigens, tumor-infiltrating immune cells and tumor mutational burden (TMB), however none have proven good enough to tailor therapy.

Efficacy of antineoplastic treatment is determined by its direct effects on cancer cells, but off-target effects on the host immune system may contribute as well (6, 7). The immune system is a key player in the progression as well as containment of cancer, and new treatment strategies based on ICI target this interaction (8). Cytokines are major regulators of the immune system as they are key mediators for both defense against infectious pathogens as well as neoplasms (9–11). Contrary to our extensive understanding of intracellular resistance mechanisms to chemotherapy there is a knowledge gap on the interaction between immune function and antineoplastic therapy and how this interaction impacts treatment efficacy, resistance, toxicity and patient outcome.

Previous studies assessing the immune system in NSCLC have reported associations between circulating (plasma/serum) levels of cytokines (interleukins, TNF-α and IFN-γ) (12–15), proinflammatory proteins (CRP, LDH) (12, 16) or mRNA encoding IFN-γ (17) and efficacy, response, progression-free survival and overall survival. However, results on circulating levels of signaling-molecules mostly reflect a “spill-over” from local sites with inflammation or immune activation. Thus, this relatively simple approach lacks information about immune function *per se* and its complex framework of interactions. Traditionally, the study of immune-signaling-pathways has been conducted on purified cells, cultured cell lines or through circulating levels of cytokines. However, *ex vivo* immune response in whole blood involves multiple immunological signaling pathways between different cell types to establish an inflammatory microenvironment (18). This emphasizes the importance of immunologic analyses reflecting the natural microenvironment.

Functional characterization of the immune system could gain more dynamic information as compared to the conventional static analyses of circulating levels of signaling molecules and cells. Assessing immune function could furthermore reveal critical information about the immune response to therapeutic interventions, revealing predictive biomarkers for tailored care and precision medicine. However, use of functional immunoassays in clinical practice has previously been limited due to challenges in standardizing sample preparation and assays, and hence output interpretation (19).

TruCulture^®^ is a recently developed, standardized immunoassay, revealing the induced immune response as a proxy for immune function. It is based on whole-blood *ex vivo* stimulation of innate immunologic signaling pathways through, among other, Toll-like receptors (TLRs) (18, 20, 21). The focus on TLRs are pertinent as these are increasingly recognized as regulators of tumor-promoting inflammation and promoters of tumor survival signals (22). Recent studies applying TruCulture^®^ have characterized immune function by this assay in association with e.g. Type 1 diabetes (23), schizophrenia (24), tuberculosis (25), pancreaticoduodenectomy (26), hematopoietic stem cell transplantation (27), neuroborreliosis (28), psoriasis (29) and in healthy individuals (18, 20, 21, 23–26).

The aim of this study was to investigate the innate immune function by use of TruCulture^®^ in patients with newly diagnosed inoperable NSCLC before and during treatment with ICI, chemotherapy (CT) or chemo-radiotherapy (CT/RT). This were done by examining the cytokine response to pathogens through extracellular (TLR1/2/4/6, Dectin-1) and intracellular (TLR3, TLR7/8) signaling pathways of the innate immune system. We hypothesized that baseline immune function as well as changes during treatment differ among patients receiving ICI or CT and associates with 12-month survival.

## Material and methods

### Study design

Prospective non-randomized study of patients with newly diagnosed advanced or locally advanced NSCLC referred for treatment with ICI, CT or chemo-radiotherapy (CT/RT). The study is part of an interdisciplinary prospective observational trial, Immune-Mo (Immune function in patients undergoing immune modulating interventions), conducted in collaboration with PERSIMUNE, Centre of Excellence for Personalized Medicine of Infectious Complications in Immune Deficiency. The trial was approved by The Regional Ethics Committee (H-18042903, H-17024315) and Data Protection Agency (RH-2015-04, I-suite03 605). All patients provided written informed consent.

### Participants

Patients (male and female), newly diagnosed with NSCLC stage IIIA-IV, were eligible for the study and ineligible if they had a synchronous cancer, pregnancy, chronical inflammatory disease, a poor performance status (>2) or previously received same antineoplastic treatment. Staging was performed according to the 8th edition of TNM (American Joint Committee on Cancer and Union for International Cancer Control TNM staging) (30). Patients were recruited from the Dept. of Oncology, Rigshospitalet from March 2018 to April 2019. Treatment was given according to the Danish National Guidelines - with either chemotherapy or immunotherapy (PD-L1 expression >50%) (31). Follow-up time was minimum 12 months after inclusion or death, whichever came first. The following variables were collected from patient files (**Table 1**): Age, sex, smoking (present/former/never), histology, stage, PD-L1 expression determined by the Tumor proportion Score (TPS) (on PD-L1 IHC 22C3 pharmDx assay), co-morbidities and performance status (PS); Treatment data including chemotherapy, radiotherapy (type and timing), immune checkpoint inhibitor and the use of steroids in proximity to blood sampling time points; Patient outcome: Response according to RECIST v. 1.1. on computerized tomography (CT) following three months of treatment and 12-month survival.

**Table 1:** Patient characteristics for all patients, patients treated with chemotherapy (palliative chemotherapy or concurrent chemoradiotherapy), and palliative immunotherapy.

	All patients n=37 (%)	Chemotherapy n=24 (%)	Immune checkpoint inhibitor (ICI*) n=13 (%)
Median age	68 years (46-81 years)	67 years (47-81 years)	70 years (58-81 years)
Gender (female (%))	21 (57%)	11 (46%)	10 (77%)
**Tobacco-use**			
Ever (n (%))	16 (43)	12 (50)	4 (31)
Former (n (%))	20 (54)	11 (46)	9 (69)
Never (n (%))	1 (3)	1 (4)	0 (0)
**Histology**			
Squamous (n (%))	8 (22)	7 (29)	1 (8)
Non-squamous (n (%))	28 (76)	17 (71)	12 (92)
Other (n (%))	1 (3)	0 (0)	
**Stage**			
IIIA (n (%))	1 (3)	1 (4)	0 (0.0)
IIIB (n (%))	11 (30)	9 (38)	2 (15)
IV (n (%))	25 (68)	14 (58)	11 (85)
**PDL-1 expression**			
> 50% (n (%))	11 (30)	0 (0)	11 (84)
1-50% (n (%))	18 (49)	17 (71)	1 (8)
< 1% (n (%))	7 (19)	6 (24)	1 (8)
unknown (n (%))	1 (3)	1 (4)	
**PS**			
PS 0 (n (%))	17 (46)	13 (54)	4 (31)
PS 1 (n (%))	15 (40)	8 (33)	7 (54)
PS 2 (n (%))	5 (14)	3 (13)	2 (15)
**Treatment**			
Chemotherapy (n (%))	19 (51)	19 (79)	0 (0)
ICI* (n (%))	13 (35)	0 (0)	13 (100)
CT/RT** (n (%))	5 (14)	5 (21)	0 (0)
Palliative RT*** (n (%))	7 (19)	5 (21)	2 (15)
Glucocorticoids (n (%))	7 (19)	6 (25)	1 (8)

(*ICI=Immune checkpoint inhibitor, **CT/RT=chemo-radiotherapy, ***RT=radiotherapy).

### Blood sampling

Consecutive blood sampling was conducted as follows: Within 1 day of starting therapy (Day 0), during treatment at day 7, 21 and 80 for CT-treated patients (CT and CT/RT) and day 21 and 80 for ICI-treated patients (ICI). Day 21 was prior to the second treatment cycle (ICI or CT) and before initiation of concurrent radiotherapy (CT/RT); Day 80 was at the expected time of the evaluation CT scan. For the chemo-radiotherapy patients (CT/RT), the day 80 blood sample and the evaluation scan were conducted two months later (i.e. day 140) to allow for radiotherapy. Blood samples at each time point were investigated by TruCulture^®^ (see below) and standard hematology (leucocyte and differential count, hemoglobin and platelets), the latter conducted as routine analysis at an ISO-certified hospital laboratory.

### TruCulture^®^

TruCulture^®^ (Myriad RBM; Austin, Texas, USA) was conducted according to the manufacture’s recommendation. Blood was sampled in lithium heparin tubes and transferred immediately to the laboratory. One hour (± 15 min) after blood sampling, 1 mL of whole blood was aliquoted to each prewarmed TruCulture^®^ tube, inserted into a digital dry block heater (VWR International A/S, Soeborg, Denmark) and maintained at 37°C for 22 hours (± 30 min). At the end of the incubation period, TruCulture^®^ tubes were opened and a valve was inserted to separate the sedimented cells from the supernatant and stop the stimulation reaction. Liquid supernatants were aliquoted and immediately frozen at −20°C, transferred and stored at −80°C after 1-7 days until thawed for analysis. A custom designed TruCulture^®^ panel comprising four different immune stimuli and an unstimulated tube was applied: i) Resiquimod R848 (R848, imidazoquinoline compound) providing stimulation through TLR7/TLR8, mimicking viral single-stranded RNA; ii) Polyinosinic:polycytidylic acid (PolyI;C), a synthetic double-stranded RNA analogue providing stimulation through TLR3, mimicking viral double-strand RNA; iii) Bacterial endotoxin (lipopolysaccharide, LPS) from E.coli O111:B4 providing immune cell stimulation through TLR4; iv) Heat killed *candida albicans* (HKCA, whole microbe) providing a complex immunologic stimulation through TLR1/2/4/6 and Dectin-1 and v) no stimulation/NULL, containing TruCulture^®^ media without stimuli, revealing *in vivo* blood immune cell activation and circulating cytokine levels. The following cytokines, and mediators of primarily the innate immune response, were analyzed; IL-1β, IL-6, IL-8, IL-10, IL-12p40, IL-17A, TNF-α and IFN-γ in each liquid supernatant by an 8-plex Luminex (R&D Systems, BIO-Techne LTD) using a Luminex 200 instrument (LX200, R&D Systems, BIO-Techne LTD). Results are reported in pg./ml. In the following, cytokines TNF-α, IL-1β, IL-6 and IL-8 are referred to as proinflammatory cytokines; IFN-γ and IL-12p40 as anticancer cytokines; IL-10 as anti-inflammatory cytokine and IL-17A as a Th17 cytokine. An in-house reference level for TruCulture^®^ induced release of cytokines was generated upon implementation of the method and included for comparison.

### Statistical methods

Statistical analyses were performed using R (v 3.6.1). Unless otherwise noted, patients treated with CT and CT/RT was considered as one group for the analysis and patients treated with ICI as a separate group. Baseline variables were compared between patients allocated to ICI vs CT by Mann-Whitney U test. The study was planned as an explorative study, thus no power calculation was performed.

The influence of radiation and steroid treatment on TruCulture^®^ immune response was investigated by inspecting the TruCulture immune response curves after radiation therapy, respectively results of individual blood samples taken within 24 hours of steroid treatment. Williams type test was used to calculate p-values. P-values <0.05 were considered significant.

To evaluate whether blood cell counts influenced cytokine levels, each specific cytokine was compared to all other cytokines and to each specific leukocyte subpopulation cell count. As to not introduce other factors which might bias these results, only baseline data before patients received treatment was analyzed. All patients were included in this analysis. A matrix of covariances between cytokine levels at baseline (stimulated and unstimulated) and blood cell counts were calculated by Pearson’s method using the RStudio package cor. To visualize the network of correlations RStudio package Qgraph was used. A threshold for plotting correlations was set at an alpha value of p=0.05 and a correlation coefficient of >0.5 or ≤0.5. Multiple testing correction was not done due to the explorative nature of this analysis.

Variables investigated over time, i.e., TruCulture^®^ induced release of cytokines and blood cell counts were plotted by locally estimated scatterplot smoothing (LOESS), a non-parametric regression model based on k-nearest neighbors, displaying 95% confidence intervals alongside 95% confidence intervals of the reference. Linear mixed effects models under inequality constraints (CLME) were used to compare cytokine levels between; 1) Patients treated with ICI or CT and 2) patients treated with CT or CT/RT surviving or dying within 12-months follow-up. Survival was not compared within the ICI group due to low number of observations. The CLME analysis was performed by R package CLME (32) with applying constraints tested for umbrella order restrictions. CLME does not depend on normality.

A comparison of blood cell counts, and Neutrophil/Lymphocyte ratio was conducted in patients treated with chemotherapy (CT and CT/RT) stratified by vital status at 12 months. Median and interquartile range (IQR) were calculated, and p-values were calculated by Welch two sample t-test.

## Results

### Study patients

**Figure 1:**
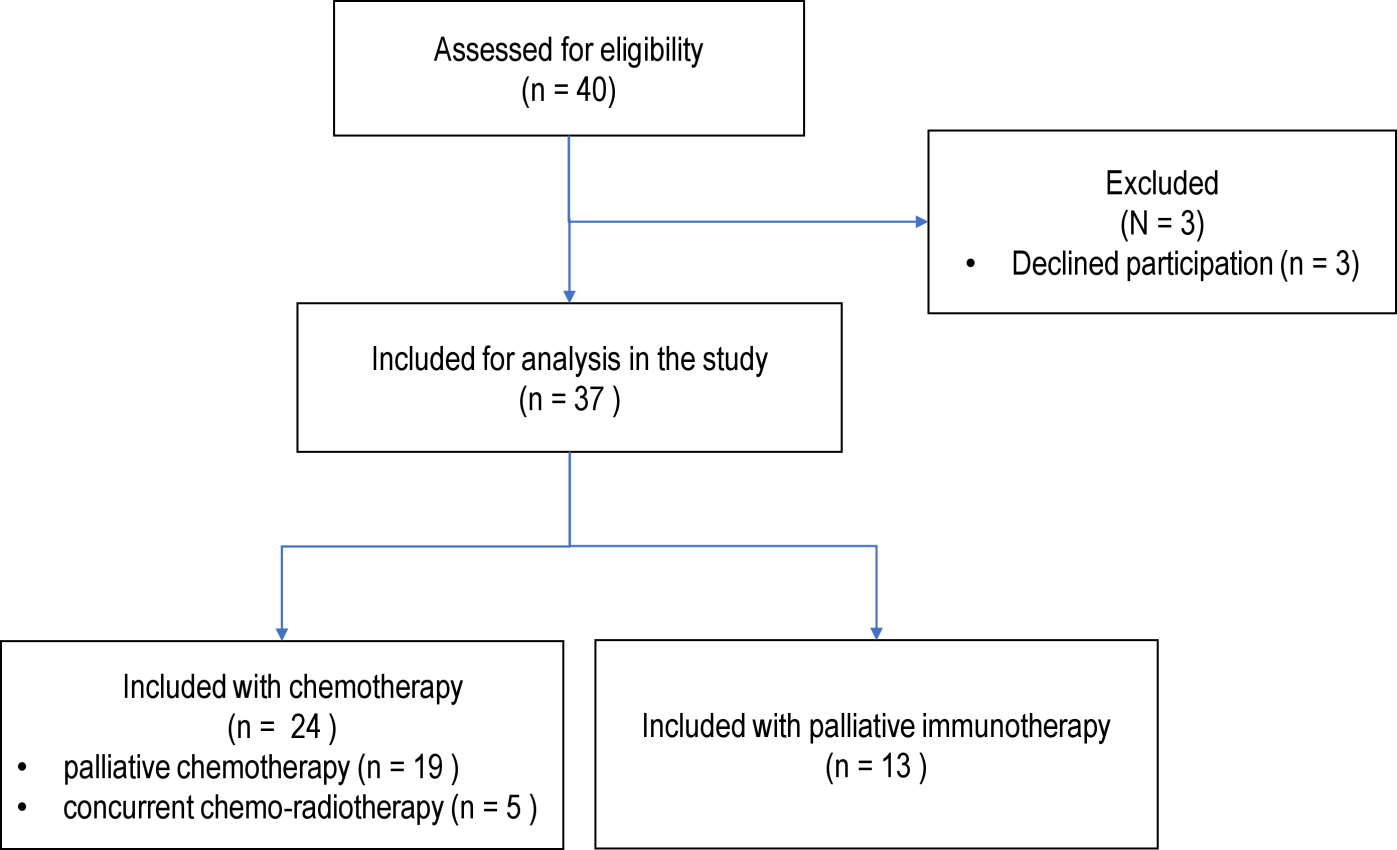
Flowchart displaying patient inclusion and exclusion in the study. All patients were assessed prior to initiation of treatment.

Forty patients with newly diagnosed NSCLC were assessed for eligibility and offered inclusion in the study, three declined (8%) (**Figure 1**). Thirty-seven patients were enrolled with a median age of 68 years (range 46-81 years), 57% were females. Patient characteristics for the 37 enrolled patients are displayed in **Table 1**.

**Figure 2:**
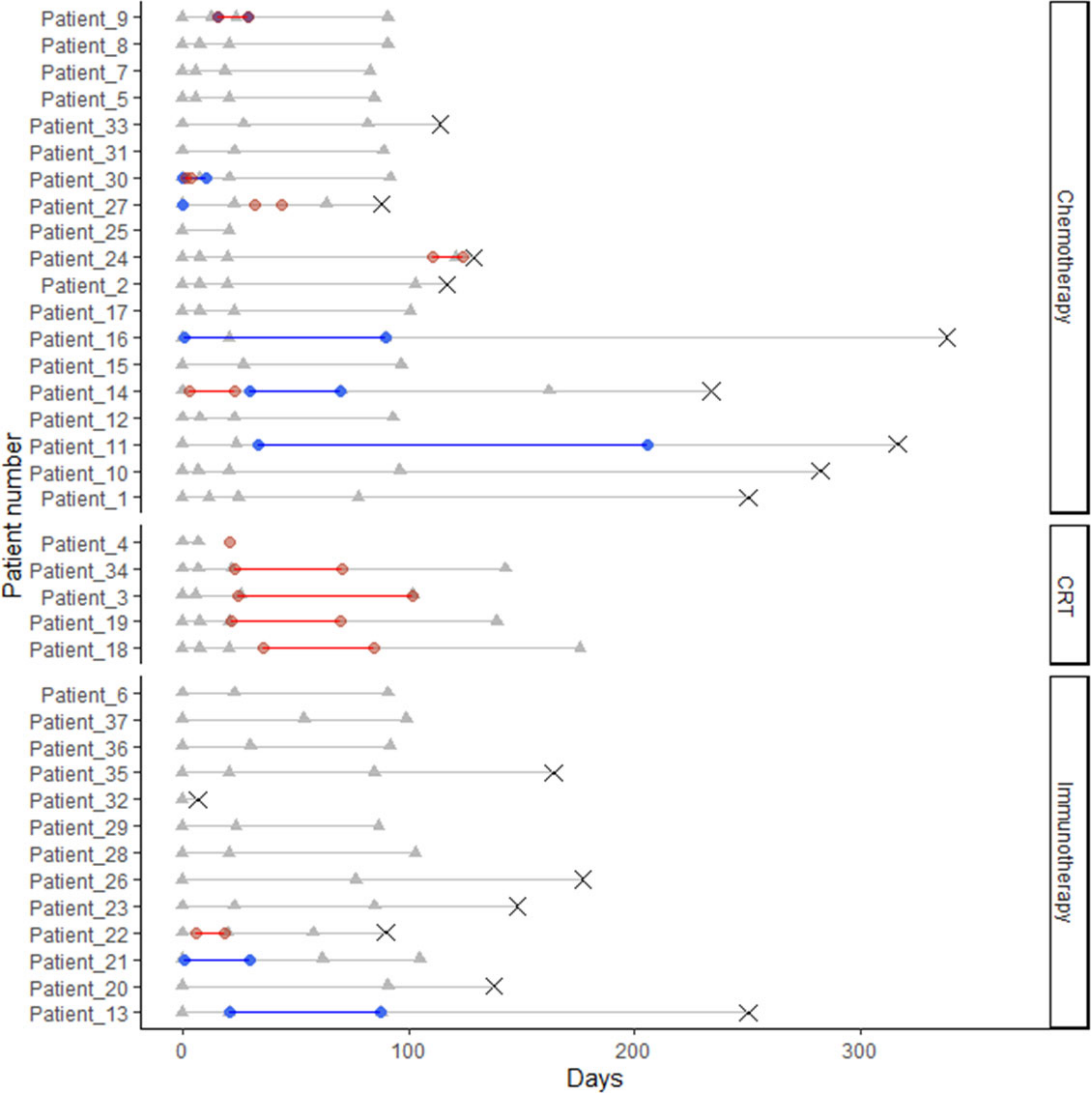
Swimmers Plot displaying timelines for all patients from admission (day 0) until 1-year follow up or death. The following events are displayed: Blood samples (grey triangles), radiotherapy (red diamonds), Glucocorticoids (blue diamonds) and death (grey X). Patients are grouped according to treatment (chemotherapy (CT), concurrent chemo- and radiotherapy (CRT) and immunotherapy (ICI).

Treatment and disease trajectory of the individual patients are depicted by a Swimmers Plot in **Figure 2**. Thirteen patients (35%) received treatment with ICI (pembrolizumab (n=12), atezolizumab (n=1)), 24 patients (65%) received CT (platin-based chemotherapy and vinorelbine) including five patients receiving concurrent chemo- and radiotherapy (CT/RT). Seven patients (19%) received palliative radiotherapy during or within a week prior to blood sample collection. Eight of the 37 patients (22%) received doses of 25-75mg corticosteroids resulting in 11 of the 119 (9%) blood samples being taken during (or within 24h after) treatment with corticosteroids.

Two patients progressed and died during treatment, resulting in 95% (35/37) patients completing initial treatment. One patient (3%) presented with clinical progression, unconfirmed by CT scan, 34 patients (92%) underwent an evaluation CT scan with a median of 78 days after treatment start: Fourteen patients (40%) had progressive disease (PD) on the first evaluation scan, twenty patients (57%) had stable disease (SD) or partial response (PR), The 12-months survival rate was 57% (21/37).

### Study blood sampling

In total, 119 out of 135 planned blood samples (88%) were performed. All 37 patients had baseline samples (day 0); 16 (66% of the CT-treated patients) had day 7, 33 (89%) had day 21 and 32 (86%) had the fourth sample (day 80). The five CT/RT-treated patients had the last sample taken day 140. The reason for incomplete blood sampling was either blood sampling being conducted at another hospital or death (two patients).

### Influence of radiation and steroids on TruCulture^®^ immune response

Neither the unstimulated nor the stimulated immune response differed between patients treated with or without radiotherapy. Thus, results are presented according to treatment with either CT (both CT and CT/RT) or ICI (**Table 1**). We were not able to statistically compare TruCulture^®^ response in samples collected outside or during steroid treatment due to the scarce number of samples collected during steroid treatment (<10%). Inspection of the unstimulated and stimulated response however revealed a tendency towards lower stimulated cytokine levels for samples taken during steroid therapy, most pronounced following R848 (TLR7/8) and LPS (TLR4) stimulation (**Supplementary Figure 1**).

### Blood cell count

**Figure 3:**
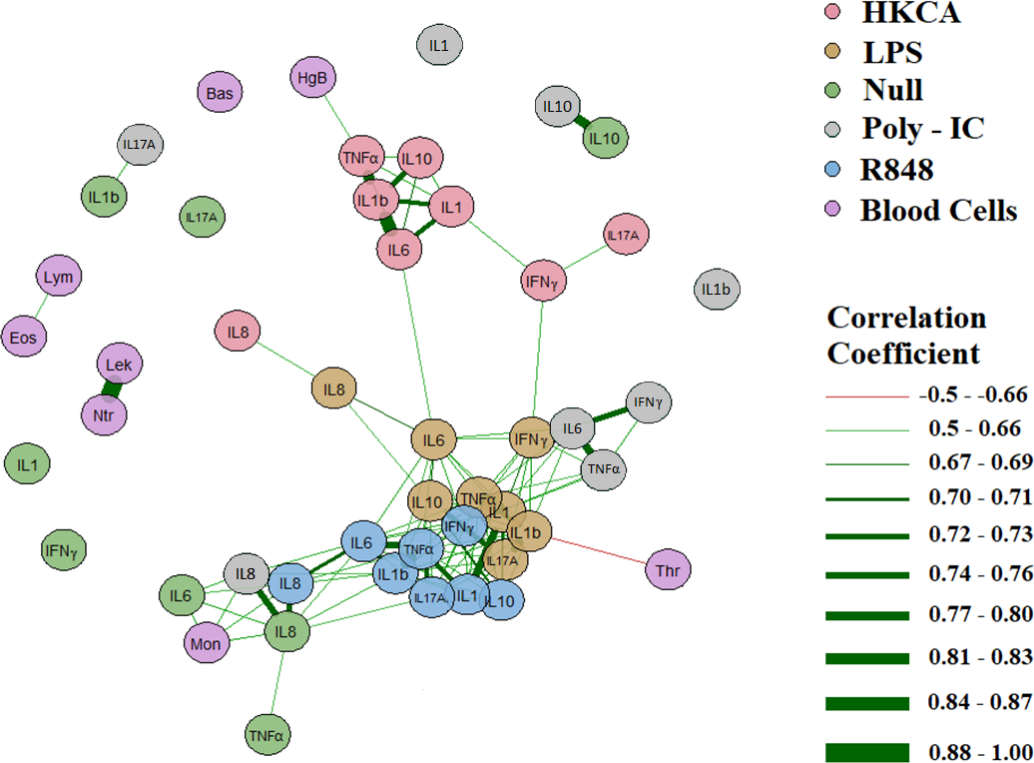
Correlation of blood cell counts and cytokines in 37 NSCLC patients based on baseline data before treatment. Only correlations with an alpha value of p=0.05 and a correlation coefficient of >0.5 or ≤0.5 are plottedas connecting lines: Green signifying a positive and red a negative correlation. Line thickness signify a correlation coefficient from -1 to 1. Mon: Monocytes, Thr: Thrombocytes, Lek: Leukocytes, Ntr: Neutrocytes, Eos: Eosinophil, Lym: Lymphocytes, Bas: Basophilocytes, HgB: Hemoglobin. All blood cells are purple, while cytokines are in different colors depending on the stimulus.

At baseline, ICI-treated patients had lower hemoglobin (p<0.001) and higher eosinophil cell count (p=0.018) than patients starting CT. There were no significant differences between ICI and CT treated patients during treatment (**Supplementary Figure 2**). Substantial and significant associations were seen between the different TruCulture^®^ induced cytokines, as expected (**Figure 3**). However, the released cytokines were only sparsely associated with blood cell counts; monocyte counts were positively associated with unstimulated IL-6 (p<0.05, correlation coefficient=0.64) and IL-8 (p<0.05, correlation coefficient=0.62) release, and positively associated with Poly I:C stimulated IL-8 (p<0.05, correlation coefficient=0.57) release and R848 stimulated IL-8 (p<0.05, correlation coefficient=0.6) release; hemoglobin was positively associated with HKCA stimulated TNF-α (p<0.05, correlation coefficient=0.50) release and platelets were negatively associated with LPS stimulated IL-1β (p<0.05, correlation coefficient=-0.50) release. We found that neither granulocytes nor other leukocyte subtypes to be associated with stimulated and unstimulated cytokine release.

### Proinflammatory cytokines (TNF-α, IL-1β, IL-6, and IL-8)

**Figure 4:**
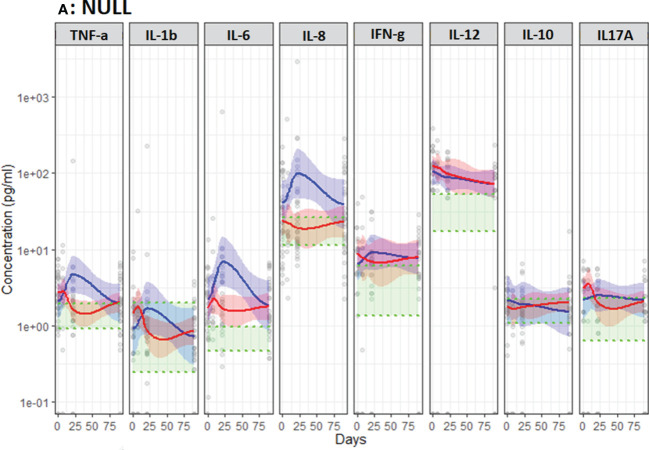
Unstimulated immune response assessed by TruCulture^®^ (NULL) in NSCLC patients during treatment with either CT (Red, n=24) or ICI (Blue, n=13). Corresponding 95% confidence interval shown as the light red respectively blue area. The shaded green area represents the in-house reference level (95% confidence interval between the green dotted lines).

**Figure 5:**
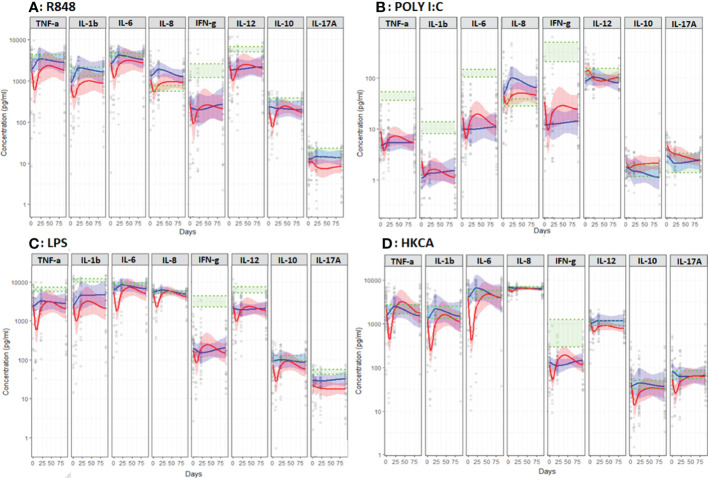
Induced immune response to different immune stimuli assessed by TruCulture^®^ in NSCLC patients during treatment with chemotherapy (CT, Red, n=24) or immunotherapy (ICI, Blue, n=13). **(A)** Resiquimod (R848), **(B)** polyinosinic:polycytidylic acid (poly I:C), **(C)** Lipopolysaccharide (LPS) and **(D)** heat-killed Candida albicans (HKCA). Corresponding 95% confidence interval shown as the light red respectively blue area. The shaded green area represents the in-house reference level (95% confidence interval between the green dotted lines).

*At baseline*, all NSCLC patients displayed higher unstimulated IL-6 and IL-12p40 and ICI-treated patients displayed higher unstimulated IL-8 compared to the healthy refence (**Figure 4**). However, Poly I:C (TLR3) stimulated TNF-α, IL-1β and IL-6 was lower in NSCLC patients compared to the reference (**Figure 5**). ICI patients displayed higher HKCA (TLR1/2/4/6 and Dectin-1) stimulated IL-8 (p=0.046 and 0.047) but lower Poly I:C (TLR3) stimulated TNF-α (p=0.022) and IL-1β (p=0.016) (**Figure 5**).

*During the first treatment cycle* with ICI, unstimulated TNF-α, IL-6 and IL-8 increased followed by a decline towards baseline levels while staying relatively stable in CT-treated patients (**Figure 4**). In patients treated with ICI unstimulated IL-8 stayed higher compared to CT-treated patients (p=0.019) (**Figure 4**). Furthermore, patients treated with ICI displayed higher levels of R848 (TLR7/8) stimulated IL-1β (p=0.019) and LPS (TLR4) and HKCA (TLR1/2/4/6 and Dectin-1) stimulated IL-8 (p=0.011 and p=0.047) (**Figure 5**).

### Anticancer cytokines (IFN-γ and IL-12p40)

*At baseline*, no differences were observed between ICI and CT-treated patients (**Figures 4**, **5**). Compared to the reference level, unstimulated IL-12p40 was higher in NSCLC patients and unstimulated IFN-γ was higher in ICI-treated patients (**Figure 4**). Across all stimuli, IFN-γ was lower and R848 (TLR7/8) and LPS (TLR4) stimulated IL-12p40 was lower in NSCLC patients compared to the reference level (**Figure 5**).

*During treatment*, no significant difference in unstimulated levels was observed between ICI and CT-treated patients. However, HKCA (TLR1/2/4/6 and Dectin-1) stimulated IL-12p40 was higher in ICI compared to CT-treated patients (p=0.018) (**Figure 5**).

### Anti-inflammatory cytokine (IL-10)

*At baseline*, no differences were observed between ICI and CT-treated patients (**Figures 4**, **5**).

*During treatment*, ICI-treated patients displayed lower and declining Poly I:C (TLR) stimulated IL-10 compared to CT-treated patients (p=0.025) (**Figure 5**). No other differences in unstimulated or stimulated IL-10 were observed comparing ICI and CT, or comparing NSCLC patients with reference levels (**Figures 4**, **5**).

### Th17 cytokine (IL-17A)

*At baseline*, no differences were observed between ICI and CT-treated patients (**Figures 4**, **5**).

*During treatment*, ICI-treated patients displayed higher LPS (TLR4) stimulated IL-17A compared to CT-treated patients (p=0.021, **Figure 5**). No other differences in unstimulated or stimulated IL-17A were observed comparing ICI and CT or comparing NSCLC patients with reference levels (**Figures 4**, **5**).

### 12-months survival in the chemotherapy group

**Figure 6:**
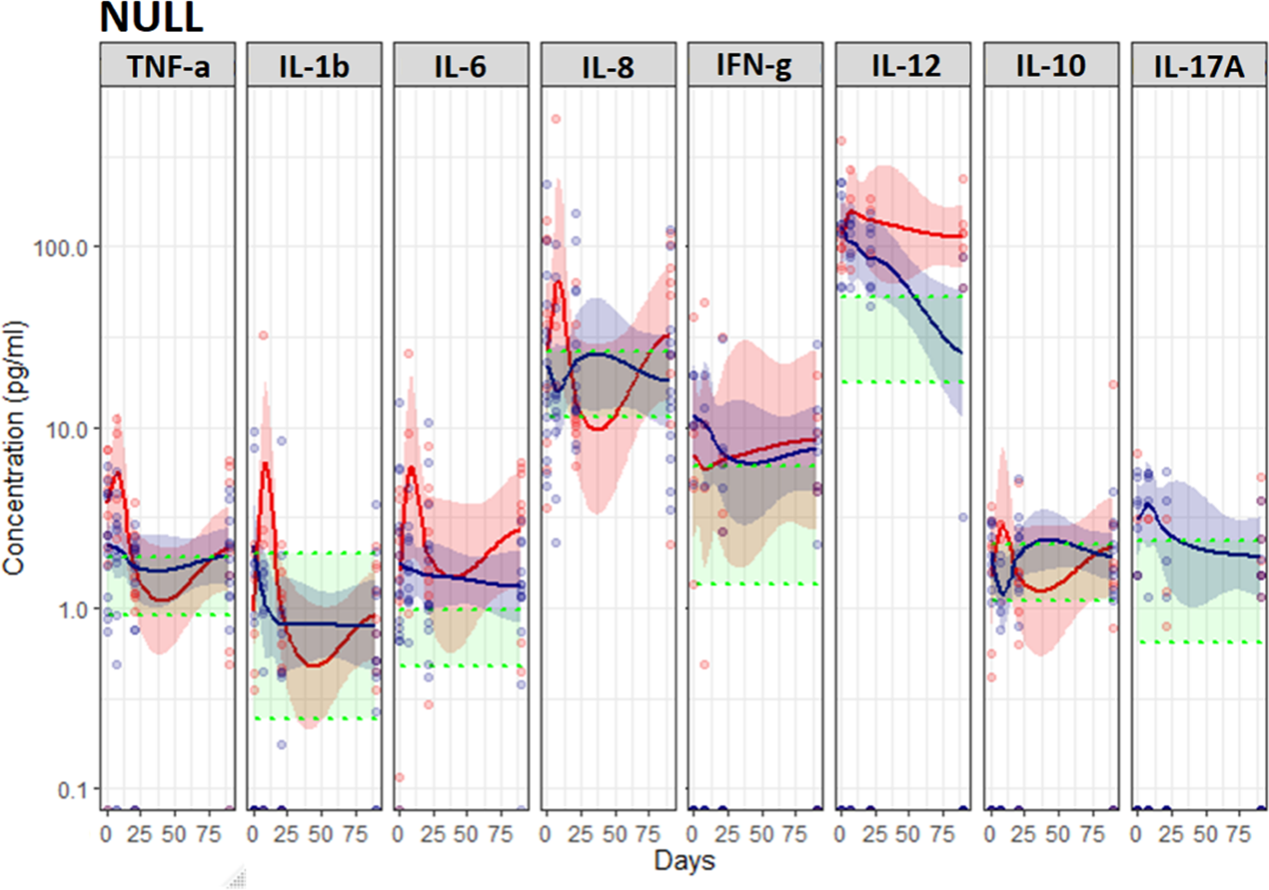
Unstimulated immune response assessed by TruCulture^®^ (NULL) in NSCLC patients During treatment with chemotherapy (CT) in patients stratified according to 12-months survival (death within 12 months (red, n=16)) and survival beyond 12 months (blue, n=8)). Corresponding 95% confidence interval shown as the light red respectively blue area. The shaded green area represents the in-house reference level (95% confidence interval between the green dotted lines). Data on IL-17a in patients treated with chemotherapy is missing due to excessive dispersion for this cytokine combined the relatively few observations causes to big uncertainty in data.

**Figure 7:**
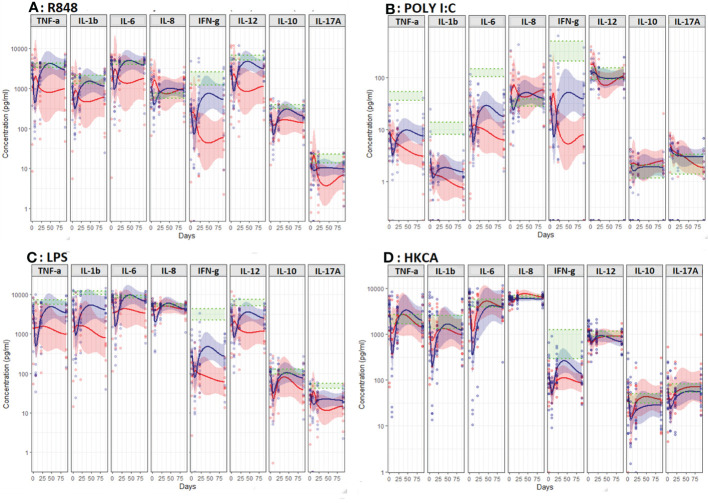
Induced immune response assessed by TruCulture^®^ during treatment in CT-treated patients stratified according to 12-months survival (death within 12 months (red, n=16)) and survival beyond 12 months (blue line, n=8)). **(A)** Resiquimod (R848), **(B)** polyinosinic:polycytidylic acid (poly I:C), **(C)** Lipopolysaccharide (LPS) and **(D)** heat-killed Candida albicans (HKCA). Corresponding 95% confidence interval shown as the light red respectively blue area. The shaded green area represents the in-house reference level (95% confidence interval between the green dotted lines).

Comparing CT-treated patients according to survival after 12 months, no significant differences in unstimulated proinflammatory, anti-cancer, anti-inflammatory or Th17 cytokines were observed at baseline (**Figure 6**). However, during therapy non-survivors displayed higher and stable unstimulated levels of the anti-cancer cytokine IL-12p40 (p=0.022, **Figure 6**) as well as lower and declining R848, LPS and HKCA stimulated IFN-γ (p=0.033, p=0.013, p=0.030) compared to survivors (**Figure 7**).

Poly I:C and LPS stimulated pro-inflammatory cytokines (IL-1β and Poly I:C stimulated IL-6) was lower and declined during treatment in non-survivors compared to survivors (p=0.005, p=0.006 and p=0.002, **Figure 7**). No differences were observed in stimulated or unstimulated IL-10 or IL-17A during treatment (**Figures 6**, **7**).

Blood cell counts, and Neutrophil/Lymphocyte ratio was analyzed in patients treated with chemotherapy (CT) stratified by vital status at 12 months. Statistical power was limited due to subgrouping of patients. However patients receiving CT who were alive after 12 months displayed significantly higher lymphocyte counts at day 0 (1.7 vs 1.0, p=0.008) and 21 (1.25 vs. 0.9, p=0.032) compared to deceased patients (**Supplementary Table 1**). Neutrophil/Lymphocyte ratio showed a similar pattern; however, it was not significantly different between the two groups (3.6 versus 6.4, p=0.099).

We did not have enough power to analyze potential associations with survival in ICI-treated patients.

## Discussion

This study demonstrates evidence of chronic innate immune activation with high unstimulated *ex vivo* release of cytokines accompanied by innate immunologic exhaustion with impaired *ex vivo* stimulated cytokine release in patients with NSCLC. This immune activation and exhaustion appeared to be alleviated during ICI, but less so during CT. Finally, CT-treated patients surviving less than 12 months displayed features of chronic innate immune activation, lower lymphocyte counts day 0 and day 21 and aggravated immune exhaustion during treatment compared to patients surviving beyond 12 months. together, this indicates a critical clinical importance of the innate immune system for the outcome in NSCLC.

The use of immunoassays for risk stratification could potentially play an important role in tailored care of cancer patients. We have demonstrated that a standardized immunoassay, TruCulture^®^, can reveal a developing immune response induced by both ICI and CT, and that in CT-treated patients, this is associated with 12-months survival. It may also be the case for ICI-treated patients, but this study did not have enough power to investigate this. TruCulture^®^ immunoassay reveals the induced immune response following *ex vivo* ligand stimulation of whole blood, allowing for standardized monitoring of cytokine release as a proxy for immune function (20, 21). We used TruCulture^®^ to screen for both extracellular (TLR1/2/4/6, Dectin-1) and intracellular (TLR3, TLR7/8) signaling pathways of the innate immune system. The present study found *higher unstimulated* and *lower stimulated* cytokine release in patients compared to the reference level. This may both reflect that cancer patients have increased immune activation *in vivo* i.e., higher circulating (unstimulated) cytokine release *and* a general immune exhaustion upon (*ex vivo*) stimulation. This pattern has previously been observed in chronically inflamed patients, e.g. in patients infected with HIV (33). Though previous studies have reported higher level of circulating cytokines in patients with NSCLC (12–16), this paper is, to the best of our knowledge, the first to describe a complex immunoassay assessing the induced immune response *ex vivo* as a proxy for immune function in lung cancer patients.

Previous studies of NSCLC-patients have reported an association between circulating levels of cytokines and response to ICI (12–16, 34). Boutsikou et al. reported *elevated* circulating proinflammatory cytokines (TNF-α, IL-1β, IL-6, IL- 8) at diagnosis and after 3 months of treatment to be associated with improved overall survival (14). Ozawa et al. reported similar results after just 7 days, but only for IL-6 (12). However, Sanmamed et al. (13) found *decreasing* circulating levels of IL-8 during treatment with ICI (2–4 weeks post-treatment) to be associated with favorable treatment response and longer overall survival in patients with melanoma and NSCLC. Also, Schalper et al. found elevated baseline serum levels of IL-8 to be associated with poor overall survival (34).

A possible explanation for the discrepancy in these results is the difference in the timing of the analyses; our results for unstimulated pro-inflammatory cytokines – most closely mimicking circulating levels - suggest an increase during the first three weeks of treatment with ICI followed by a decrease (**Figure 4**). Combined, these results also indicate that ICI improves the stimulated innate immune response as observed *ex vivo* in the present study (**Figure 5**). Given the association between this and improved survival, TruCulture^®^ may be a valuable immunomonitoring tool for early assessment of response to ICI in NSCLC and other cancers. However, this finding requires confirmation in larger studies. Furthermore, considering the extensive fluctuations in immune function - higher unstimulated IL-12p40 and lower stimulated proinflammatory and anticancer cytokines day 21 in non-survivors - timing of blood sampling and in-depth knowledge on *ex vivo* stimulation vs. circulating levels is essential for accurate interpretation of immunomonitoring results.

Higher lymphocyte counts and lower Neutrophil/Lymphocyte ratio were observed in patients surviving beyond 12 months. The latter was, however, not significant, in contrast with previous findings (27). This is most likely due to the limited power in this study. The finding, however, of a positive association between higher lymphocyte count and survival, emphasizes the importance of cells of the adaptive immune system for disease progression in cancer patients.

We have previously reported on associations between TruCulture induced cytokine release and different leucocyte subpopulations in Gjaerde et al. (27). In this study, we found that “increases in cytokines were not merely caused by an increase in the number of leucocytes”, as we only found few associations between different leukocyte subpopulation counts and the magnitude of induced cytokine release. In the present study, we found monocyte counts to be associated with stimulated and unstimulated IL-8 and IL-6 release, emphasizing the potential critical role of these signaling molecules for differentiation of monocytes (IL-8) to macrophages (IL-8 and IL-6). As we found neither granulocytes nor other leukocyte subtypes to be associated with neither stimulated nor unstimulated TruCulture^®^ cytokine release, our results are in accordance with those by Gjaerde et al. (27), namely that TruCulture^®^ may reveal an improved function of the immune cells and not simply increased immune cells in cancer patients. However, it should be emphasized that our study and hence results are explorative and needs confirmation in larger data sets.

IFN-γ is produced predominantly by activated T lymphocytes, NKT cells and natural killer (NK) cells and play an important role in host defense against pathogens as well as regulating tumor development (35). Furthermore, it is an important activator of macrophages and inducer of major histocompatibility complex class II molecule expression. Studies have found high circulating IFN-γ levels to be predictive of a good treatment response as well as overall survival in NSCLC (14, 17). Consistent with our results, high immunologic capacity to produce IFN-γ is therefore likely to be beneficial for the clinical outcome of lung cancer patients, with a lower *ex vivo* stimulated response predicting a poor outcome. We infer that the chronic innate immune activation and exhaustion that ICI treated patients had higher levels of both stimulated and unstimulated IL-8 levels compared to CT treated patients. As IL-8 mediated effect on survival is independent of PD-L1 positivity (34), the inclusion criteria for ICI (PD_L1 expression) seems not to be responsible for the opposing levels of both stimulated and unstimulated IL-8 observed between the two treatment groups during treatment.

Consistent with the study by Schalper et al. investigating baseline serum levels of IL-8 in ICI treated patients (34), we found elevated *unstimulated* baseline IL-8 levels to be associated with poor prognosis in CT treated patients. The same study found the IL-8 mediated effect on survival independent of PD-L1 positivity (criteria for frontline ICI treatment). In the present study, patients treated with ICI displayed higher levels of both *stimulated* and *unstimulated* IL-8, compared to CT treated patients. We also found the opposing levels of both *stimulated* and *unstimulated* IL-8 observed between the two treatment groups, to be associated with monocyte counts. Differences in the two treatment groups at baseline might reflect a different activation and response of these cells and might be part of the mechanisms of fighting cancer and thereby achieve a better outcome.

Our study is explorative and limited by the relatively low number of study participants and a heterogenous patient population regarding treatment and stage combined with the large number of variables generated by the TruCulture^®^ set-up. A larger study could reveal more subtle changes in the functional immune responses and allow analysis of survival data for the ICI-treated patients. As the study focused on the extra- and intracellular immunological signaling pathways of the innate immune response, information of the adaptive immune system was not as thoroughly investigated. A strength of the study was however the use of a highly standardized immunoassay allowing for technology transfer and ensuring validity in repeat studies.

Potential confounders were treatment with glucocorticoids and radiotherapy which both influence the immune system (36–39). Glucocorticoids has a known suppressing effect on the immune system (37), and we did observe a tendency towards reduced stimulated cytokine response in samples collected during periods with glucocorticoid treatment (**Supplementary Figure 2**). However, only 11/119 blood samples were collected during treatment with glucocorticoids, and the effect on the overall results is likely negligible. Previous studies have suggested that radiotherapy both suppress and stimulate the immune system; with paradoxical effects of stimulation or immunosuppression in different scenarios of radiotherapy (36, 37). A total of 11 patients received radiotherapy during the study period: five concurrently with chemotherapy. We did not observe any significant difference between this group and patients not receiving radiotherapy.

Patients in this study displayed a lower stimulated response compared to the reference level, which is an in-house reference and thus neither age and gender matched, nor matched for use of tobacco, alcohol, or immune-modulating medication with patients included in the present study. Results from the reference level are therefore not adjusted for potential confounders. As studies have found an association between aging and a decline in both adaptive and innate immune responses, but paradoxically also an association with a state of chronic inflammation (40), part of the observed differences in immune function may be caused by the aging in our study subjects. Although an in-house reference level adjusted for potential confounders would have strengthened our results and is preferable for future studies, our findings of an exhausted immune system unable to act properly towards pathogens (and theoretically cancer cells) still indicates a possible immune deficiency or exhaustion/anergy as part of the development of cancer. This is further supported by the well-established evasion of immune surveillance as one of the hallmarks of cancer (41).

## Conclusion

This study demonstrated chronic innate immune activation and exhaustion prior to treatment in patients with NSCLC. These characteristics seemed to be alleviated during treatment with ICI, but not chemotherapy. Furthermore, CT-treated patients with survival less than 12 months displayed features of chronic innate immune activation with low lymphocyte counts and exhaustion during and after treatment, emphasizing the clinical importance of the innate immune system for outcome in NSCLC. Thus, these findings indicate that the immune system in cancer patients may become exhausted due to chronic activation and proinflammation, and furthermore that a high level of immune activation and exhaustion is associated with increased mortality, which may be reversed by ICI. Future studies are warranted to confirm these findings.

## Data availability statement

The original contributions presented in the study are included in the article/**Supplementary Material**, further inquiries can be directed to the corresponding author/s.

## Ethics statement

The studies involving human participants were reviewed and approved by The Regional Committee of the Capital Region of Denmark. The patients/participants provided their written informed consent to participate in this study.

## Author contributions

SN, JL, SO, and BF designed and conceptualized the study. Data collection and primary analysis was done by HR and KE, supervised by MP, SL, SO, and BF. All authors contributed to the article and approved the submitted version.
