# Supplementary material for: Innate immune function during antineoplastic treatment is associated with 12-months survival in non-small cell lung cancer
Source: Front Immunol. 2022 Dec 12;13:1024224. doi: 10.3389/fimmu.2022.1024224 (PMC9791214; doi:10.3389/fimmu.2022.1024224)
Supplement: Supplementary file 1 [file DataSheet_1.docx]

Supplementary Material

|  | **Baseline Day 0** | | | **Day 21** | | | **Day 90** | | |
| --- | --- | --- | --- | --- | --- | --- | --- | --- | --- |
|  | **Alive >12 months  (n=10)** | **Dead >12 months (n=9)** |  | **Alive >12 months (n=10)** | **Dead >12 months (n=9)** |  | **Alive >12 months (n=9)** | **Dead >12 months (n=9)** |  |
|  | **Median (Q1-Q3)** | **Median (Q1-Q3)** | **p-value** | **Median (Q1-Q3)** | **Median (Q1-Q3)** | **p-value** | **Median (Q1-Q3)** | **Median (Q1-Q3)** | **p-value** |
| **Hemoglobin** | 8.6  (8.4-9.3) | 8.4  (7.5-8.6) | 0.5 | 7.7  (7.4 -8.2) | 7.5  (7.5-7.7) | 0.6 | 7.2  (6.2-7.4) | 6.4  (5.9 – 7.2) | 0.3 |
| **Thrombocytes** | 270  (235-314) | 324  (318-358) | 0.4 | 225 (144-261) | 310 (250-357) | 0.3 | 236 (176-308) | 290 (236-398) | 0.4 |
| **Leukocytes** | 8.8  (7.4-9.4) | 8.4  (7.1-11.9) | 0.3 | 5.7 (3.0-6.7) | 4.3  (2.7-7.2) | 0.2 | 5.3  (4.3 – 6.3) | 10.1  (5.1-11.5) | 0.081 |
| **Neutrophils** | 5.7  (4.8 – 6.5) | 6.2  (5.6-9.5) | 0.13 | 3.3  (1.3-4.6) | 4.3  (2.7-7.2) | 0.10 | 3.2  (1.9- 4.0) | 9.0  (2.9-9.2) | 0.87 |
| **Eosinophils** | 0.1  (0.03-0.3) | 0.05  (0.0-0.1) | 0.11 | 0.0  (0.0-0.075) | 0.0  (0.0-0.02) | 0.6 | 0.05  (0.0-0.1) | 0.0  (0.0-0.1) | 0.9 |
| **Basophils** | 0.0  (0.0-0.1) | 0.0  (0.0-0.04) | 0.49 | 0.0  (0.0-0.0) | 0.0  (0.0-0.01) | 0.7 | 0.0  (0.0-0.0) | 0.0  (0.0-0.0) | 0.4 |
| **Lymphocytes** | 1.7  (1.2-2.1) | 1.0  (0.7 1.2) | 0.008 | 1.25  (1.0-1.7) | 0.9  (0.8-1.2) | 0.032 | 1.3 (0.98-1.9) | 0.9  (0.7-1.2) | 0.10 |
| **Monocytes** | 0.7  (0.5-0.8) | 0.7  (0.4 (0.7) | 0.44 | 0.45 (0.3-0.83) | 0.6  (0.5-0.9) | 0.3 | 0.6  (0.5-0.6) | 0.6  (0.5-0.7) | 0.6 |
| **Neutrophil / Lymphocyte ratio** | 3.6 (2.7-4.2) | 6.4  (4.4-10.6) | 0.099 | 1.7  (1.2-2.9) | 5.8  (2.7-7.3) | 0.11 | 2.3 (1.8-2.9) | 7.7  (2.2-16.5) | 0.078 |

**Supplemental Table 1** Hematology during treatment day 0, day 21 and day 90 in NSCLC patients treated with CT stratified by vital status at 12 months.

| 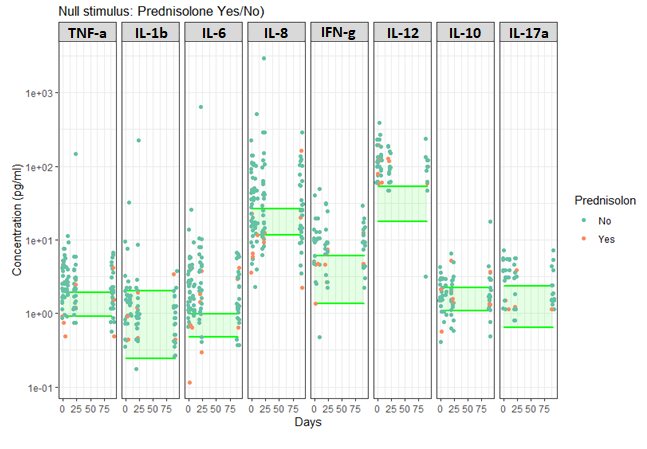 |  |
| --- | --- |
| 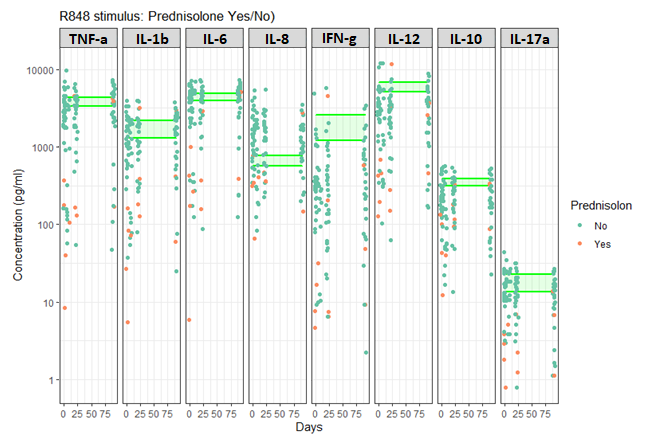 | 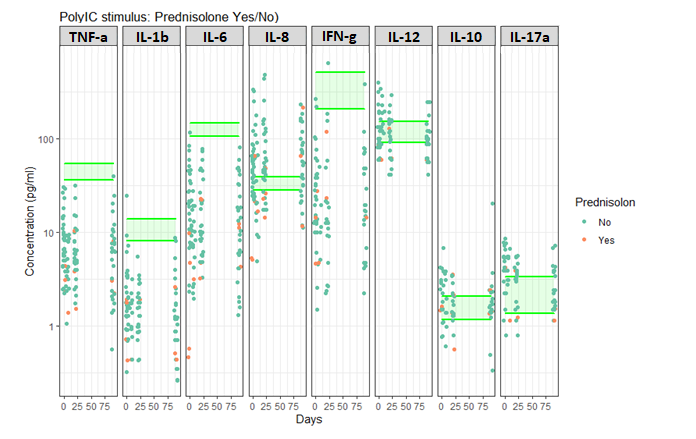 |
| 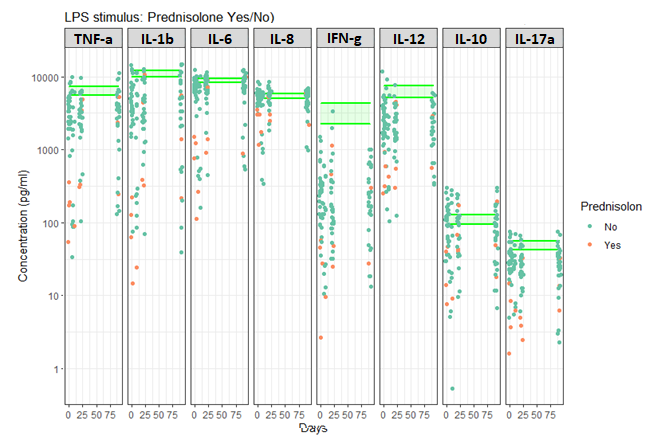 | 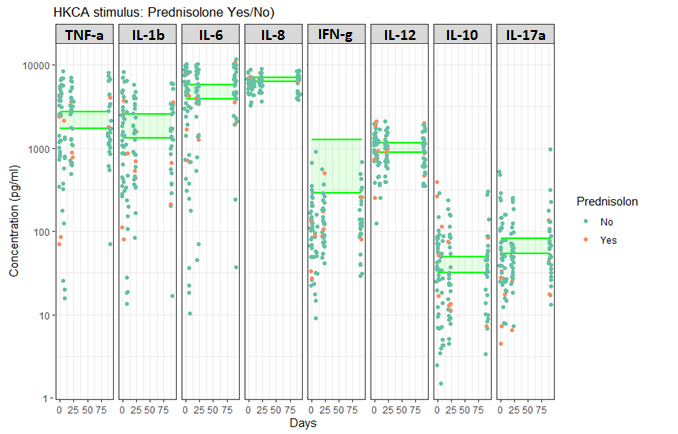 |

**Supplementary Figure 1:** The influence of steroids on TruCulture® immune response in patients with NSCLC during treatment with CT or ICI. Displaying cytokine response in samples collected during (red, n=7) or outside (green, n=30) steroid treatment. A) Resiquimod (R848), B) polyinosinic:polycytidylic acid (poly I:C), C) Lipopolysaccharide (LPS) and D) heat-killed Candida albicans (HKCA). The shaded green area represents the in-house reference level (95% confidence interval between the green dotted lines).

**Supplementary Figure 2:** Hematology in NSCLC patients during treatment day 0, day 7 (CT-treated patients), day 21 and day 80. Chemotherapy treated patients (red n=24) and ICI patients (blue, n=13) with 95% confidence interval in the shaded area. A) leukocytes; B) neutrophils; C) monocytes; D) lymphocytes; E) Eosinophils; F) Basophils; G) Hemoglobin and H) Platelets. The shaded green area represents a healthy reference level (95% confidence interval between the green dotted lines).
